# Current situation and challenges for mental health focused on treatment and care in Japan and the Philippines - highlights of the training program by the National Center for Global Health and Medicine

**DOI:** 10.1186/s12919-020-00194-0

**Published:** 2020-08-03

**Authors:** Crystal Amiel Estrada, Masahide Usami, Naoko Satake, Ernesto Gregorio, Cynthia Leynes, Norieta Balderrama, Japhet Fernandez de Leon, Rhodora Andrea Concepcion, Cecile Tuazon Timbalopez, Noa Tsujii, Ikuhiro Harada, Jiro Masuya, Hiroaki Kihara, Kazuhiro Kawahara, Yuta Yoshimura, Yuuki Hakoshima, Jun Kobayashi

**Affiliations:** 1 Department of Environmental and Occupational Health, College of Public Health University of the Philippines Manila SEAMEO TROPMED Philippines Regional Centre for Public Health, Hospital Administration, Environmental and Occupational Health, Manila, Philippines; 2 Department of Child and Adolescent Psychiatry, Kohnodai Hospital, National Center for Global Health and Medicine, Ichikawa, Japan; 3 Department of Psychiatry, National Center Hospital of Neurology and Psychiatry, Kodaira, Japan; 4 Department of Health Promotion and Education, College of Public Health University of the Philippines Manila, SEAMEO TROPMED Philippines Regional Centre for Public Health, Hospital Administration, Environmental and Occupational Health, Manila, Philippines; 5 College of Medicine, University of the Philippines Manila, Manila, Philippines; 6 West Visayas State University Medical Center, Iloilo, Philippines; 7 Lung Center of the Philippines, Quezon City, Philippines; 8 National Center for Mental Health, Mandaluyong City, Philippines; 9 Department of Neuropsychiatry, Kindai University Faculty of Medicine, Osakasayama, Osaka, Japan; 10 Office of Social Work Service, Kohnodai Hospital, National Center for Global Health and Medicine, Ichikawa, Japan; 11 Department of Psychiatry, Tokyo Medical University, Tokyo, Japan; 12 Department of Neuropsychiatry, Kanazawa Medical, Ishikawa, Japan; 13 Department of Neuropsychiatry, Faculty of Life Sciences, Kumamoto University, Kumamoto, Japan; 14 Department of Global Health, Graduate School of Health Sciences, Faculty of Medicine, University of the Ryukyus, Okinawa, Japan

**Keywords:** Child, Mental health, Training programs, Health promotion, Japan, Philippines

## Abstract

**Background and purpose:**

Mental health has emerged as an important public health concern in recent years. With a high proportion of children and adolescents affected by mental disorders, it is important to ensure that they are provided with proper care and treatment. With the goal of sharing the activities and good practices on child and adolescent mental health promotion, care, and treatment in Japan and the Philippines, the National Center for Global Health and Medicine conducted a training program on the promotion of mental health focused on treatment and care in Japan and the Philippines in September and November 2019.

**Key highlights:**

The training program comprised of a series of lectures, site visits, and round table discussions in Japan and the Philippines. The lectures and site visits focused on the current situation of child and adolescent psychiatry, diagnosis of childhood mental disorders, abuse, health financing for mental disorders, pharmacotherapy, psychotherapy, and disaster child psychiatry in both countries. Round table discussions provided an opportunity to explore the similarities and differences between the two countries in terms of the themes discussed during the lectures.

The training program identified the need to collaborate with other professionals to improve the diagnosis of mental disorders in children and adolescents and to increase the workforce capable of addressing mental health issues among children and adolescents. It also emphasized the importance of cooperation between government efforts during and after disasters to ensure that affected children and their families are provided with the care and support that they need.

## Introduction

### Current situation of mental health in the Western Pacific region

Globally, an estimated 10 to 20% of children and adolescents are affected by mental health problems [1], with more than half occurring before the age of 14 [2]. In the Western Pacific Region, mental disorders rank third in the leading causes of disability-adjusted life years (DALY) among children [3] and the prevalence of suicide attempts is high [4]. Nevertheless, despite these alarming statistics, the figures may still be underreported due to stigma and taboo which affect help seeking and reporting of mental health problems.

The Mental Health Action Plan of the World Health Organization highlighted the importance of mental health promotion especially in the early stages of life [5]. Association of South East Asian Countries (ASEAN) countries have reported that mental health education towards students was focused on coping skills whereas teacher training focused on mental illness knowledge and how to provide support to students. Despite these mental health education thrusts, there is limited medical and psychological care available in schools, thus leading to an increased interest in creating an environment that can provide mental health support to students [6].

### Background and aim of the training program

Mental health has emerged as an important public health concern in recent years. In the Philippines, the Philippine Mental Health Act came into force in 2019, and it is expected that the general public will be more concerned about mental health services and rights of patients and their families. However, there are only five government hospitals with psychiatric facilities for children, 84 general hospitals with psychiatric units, 46 outpatient facilities, and only 2.0 mental health professionals per 100,000 people [7].

The population of the Philippines is estimated to be at 100,981,437 [8]. Over the past 20 years, infant mortality has decreased [9] and about a third of the entire population are under 14 years old [10]. About 27% of children under 5 years are malnourished.

Children with mental health problems are also a cause of concern in the Philippines [11]. An assessment of the Philippine mental health system reported a 16% prevalence of mental disorders among children [12]. In addition, the latest Global School-based Student Health Survey found that 16.8% of students aged 13 to 17 attempted suicide one or more times during the 12 months before the survey [13]. More recent initiatives on establishing the landscape of mental health problems include a nationwide mental health survey being conducted by the Department of Health. This is the first nationwide baseline study that will establish the prevalence of mental disorders in the Philippines. The study is ongoing and the results will be made available by the end of the year.

Despite mental health problems being a cause of concern among children and adolescents in the Philippines, health facilities and human resources for mental health remain limited. Currently, there are only 60 child psychiatrists in the Philippines, with the majority practicing in urban areas such as the National Capital Region. In addition, there are only 11 inpatient and 11 outpatient facilities for children and adolescents, while only 0.28 beds in the mental hospitals are allocated for children and adolescents [7]. With the focus on mental health increasing in the Philippines, it is expected that the medical treatment and mental health promotion needs of children and adolescents in the Philippines will increase in the future.

In Japan, the National Center for Global Health and Medicine (NCGM) shares the Japanese experience in promoting public health practices and medical technology advancement to developing countries. In conjunction with this, the Department of Psychiatry and Child and Adolescent Psychiatry of Kohnodai Hospital conducts training programs focusing on child and adolescent health. In 2017, Kohnodai Hospital co-created a training program for children’s mental health in disaster-affected areas in the Philippines [14]. Continuing its thrust on improving child and adolescent mental health, Kohnodai Hospital conducted another training program in partnership with the Philippine Society for Child and Adolescent Psychiatry. This training focused on identifying the current situation and challenges for the promotion of mental health focused on treatment and care in Japan and the Philippines.

The objective of this training was to share the activities and good practices on child and adolescent mental health promotion, care, and treatment in Japan and the Philippines through a series of field visits and discussions. In addition, the training aimed to create a multi-institutional network for childcare such as medical care, health, education, as well as a network of medical staff of various types of occupations between the two countries.

## Outline of the training program

### Training content

The current program was composed of a training in the Philippines and in Japan. The first training was conducted in Manila, Philippines from September 11 to 13, 2019 (Table 1). Seven Japanese mental health professionals, one social worker, and one public health researcher were dispatched to the Philippines as part of the program. The Japanese experts, engaged in providing mental health promotion, care, and management to children and adolescents, discussed with Philippine experts common mental health issues, diagnostic techniques, and practices and protocols. In addition, site visits to mental health facilities in the Philippines were conducted as part of the program.

**Table 1:** Training Program Activities in Manila, Philippines

Activity	Title	Venue
Lecture	Current situation of Child and Adolescent Psychiatrists (CAP) in Japan	Manila, Philippines
	Current situation of Child and Adolescent Psychiatrists (CAP) in the Philippines	
	Pharmacotherapy for children in Japan	
	Pharmacotherapy for children in the Philippines	
	Child diagnosis in Japan	
	Child diagnosis in the Philippines	
	Disaster child psychiatry in Japan	
	Disaster child psychiatry in the Philippines	
	Child abuse in Japan	
	Child abuse in the Philippines	
Roundtable discussion	Comparison of the current situation of child and adolescent child psychiatrists in both countries	
	Comparison of pharmacotherapy in both countries	
	Comparison of child diagnosis in both countries	
	Comparison of the current situation on disaster child psychiatry in both countries	
	Comparison of child abuse in both countries	
Site visit	National Center for Mental Health	Mandaluyong City, Philippines
	Philippine General Hospital	

The second training was held in Ichikawa, Japan from November 5 to 7, 2019 (Table 2). The participants from the Philippines - composed of four child psychiatrists and a researcher - visited government institutions providing mental health services to children and adolescents. The activities of government institutions that provide assistance related to mental health care to children and their families, including its relationship to the community, were also presented during the training.

**Table 2:** Training Program Activities in Ichikawa, Japan

Activity	Title	Venue
Lecture	Introduction to an Institution for Children’s Mental Care and Aftercare for Children	Ichikawa, Japan
	Disaster Psychiatry	
	Training for Child and Adolescent Psychiatrists in Japan	
	Child Psychiatry in a Japanese University	
	Psychologists and social workers for child mental health in Japan	
Roundtable discussion	Review of the training program in Japan	
Site visit	Ichikawa Rehabilitation Center	
	Ichikawa City Education Center	
	Ichikawa City Child Care Support Section	
	Ichikawa Child Consultation Center	

### Participants

Nine health experts from Kohnodai Hospital, National Center of Neurology and Psychiatry, and University of the Ryukyus and 31 participants coming from different Philippine health, academic, government, and non-government institutions attended the first training in the Philippines. The second training was attended by five Philippine health experts from the University of the Philippines Manila College of Medicine, College of Public Health, National Center for Mental Health, and the Lung Center of the Philippines. Table 3 summarizes the profile of the participants in both training programs.

**Table 3:** Participant profile of the training program in Manila, Philippines and Ichikawa, Japan

Country	Profession	Number
Japan	Child psychiatrist	7
	Mental social worker	1
	Professor	1
Philippines	Psychiatrist	19
	Municipal Health Officer	1
	Psychologist	1
	Psychometrician	1
	Nurse	2
	Social worker	1
	Assistant Professor	4
	Administrative Assistant	1

## Training outcome: field observations and round table discussion

### Diagnosis and prevalence of mental health problems

In Japan, increasing cases of Attention Deficit Hyperactivity Disorder (ADHD) and Autism Spectrum Disorder (ASD), *futoukou* (school refusal), and child abuse are issues of major concern. In the Philippines, child abuse, ADHD, and adjustment disorder were the top primary mental health diagnoses.

Similarities were identified in both countries in terms of trend, screening, and diagnosis of neurodevelopmental disorders. In Japan, a significant increase in cases of ADHD and ASD has been noted in recent years. In 1975, the rate of autism was recorded at 1 in 5000 but it was found to be at 1 in 100 in a more recent survey [15]. Likewise, the Philippines has reported an increase in cases of ASD, from 500,000 cases in 2008 to 1,000,000 in 2018 [16]. In both countries, initial identification of neurodevelopmental disorders is conducted in schools. When cases are identified, the schools refer the children to hospitals for diagnosis. However, the limited number of available CAPs poses a constraint.

Japan and the Philippines also identified suicide and gaming disorders as major social issues. In the Philippines, common circumstances which are correlated with mental health issues among youth are: too much academic pressure with great difficulty balancing time and excessive use of digital devices engaging in network gaming and social media. Excessive digital device and social media use can lead to depression, breakdown of personal connectedness, and cyberbullying. The Philippines, being one of the most active users of social media sites [17], is at risk of adolescent addiction and depression. In Japan, First Person Shooting (FPS) games are popular and may pose a dangerous threat to young children.

### Abuse

Psychological abuse in younger children is the most common type of abuse in Japan. Younger children experience higher rates of abuse, with most deaths due to abuse perpetrated by mothers. Neighbors were found to be the most frequent to report cases of child abuse to child counseling centers. Child abuse cases in Japan can be reported to a hotline which is available 24 h a day, 7 days a week and cases are mainly handled by the child counseling center. Currently, child counseling centers are facing difficulties in coping with rapidly increasing cases of child abuse. The number of staff in child counseling centers has increased, but the addition of child counseling centers is an urgent issue since there are few specialized hospitals that can treat children’s mental health problems such as post-traumatic stress disorder (PTSD) due to child abuse.

There are more cases of online child sexual exploitation and substance abuse in the Philippines when compared to Japan. The Philippines has been identified as one of the top sources of child pornography material [18]. While cases of antisocial behavior have been decreasing recently in Japan, the Philippines has reported that it is an emerging social issue in the country. In the Philippines, an increasing trend in sexual abuse has been observed [18]. Physical abuse is likely to be underreported because corporal punishment is a commonly accepted method of disciplining Filipino children. Psychological abuse is the least recognized and reported, even though a national baseline study found that 3 of 5 children experience it [19].

The Women and Child Protection Unit (WCPUs) provide medical and psychosocial care to abused women and children in the Philippines. Trauma-informed psychosocial processing which is based on the principle of cognitive behavioral therapy (CBT) and other therapies are utilized to treat the children brought to the CPU. There are 106 WCPUs distributed in 55 provinces across the country providing 24 h a day, 7 days a week consultation, but there is a lack of mental health professionals in all these WCPUs. Not all WCPUs have a psychiatrist, but it has been proposed to have at least one psychiatrist or psychologist for each CPU. In the meantime, social workers are being trained to process cases being handled by the CPU to address the effects of trauma. More severe cases are referred to the psychologist or psychiatrist of the unit or other government hospitals. For example, the WCPU of the National Center for Mental Health (NCMH), established in 2010, is currently headed by a general psychiatrist with two to three psychiatry residents rotating every 3 months. Most of the cases the WCPU of the NCMH cater to are victims of sexual and physical abuse.

Both countries have identified key strategies in addressing child abuse. In Japan, the national policy focused on service-oriented strategies with three key points: 1) preventing child abuse by conducting home visits; 2) early detection through a regional council for child abuse and child consultation center; and 3) by protection and independent support for abused children. Meanwhile, the Philippine Plan of Action to End Violence Against Children (PPAEVAC) focused on strengthening the administrative aspect of child abuse prevention through the following strategies: development of a national database on child abuse; conduct and utilization of relevant researches on violence against children in all settings; advocacy for laws and policies relevant to violence against children; and strengthening the capacity of Local Councils for the Protection of Children (LPCs).

Schools also play a role in preventing child abuse. The Department of Education of the Philippines has issued DepEd Order no. 40 s. 2012, also known as the Child Protection Policy. This department order describes the policy and guidelines on protecting children in school from bullying, violence, exploitation, discrimination, and abuse [20]. In Japan, when cases of abuse are discovered, the school principal handles the case.

### Human resources for mental health

The lack of child psychiatrists is common in both countries. In Japan, there are 361 accredited doctors by the Japan Child and Adolescent Psychiatric Association as of June 2019. The number of child and adolescent psychiatrists in Japan are fewer when compared with the US and Europe. As of this writing, there are only 60 child psychiatrists and eight fellows in training in the Philippines, with most of the child psychiatrists practicing in Manila. Compounding the severe lack of child psychiatrists in the Philippines is the decision of some child psychiatrists to practice their profession overseas. As a response to the inadequate number of child psychiatrists in both countries, pediatricians are being trained on how to deal with patients with depression or suicidal ideation or behaviors.

Child and adolescent psychiatrists in both countries also need to go through general psychiatry for three to 4 years before they can proceed to child and adolescent psychiatry (CAP). In Japan, there is no curriculum for CAP but the certification to practice as a CAP is being administered by the Japanese Child and Adolescent Psychiatry Society (JSCAP). The curriculum for the subspecialization of CAP in the Philippines is developed and administered by the Philippine Psychiatric Association (PAP) through the Philippine Society of Child and Adolescent Psychiatry (PSCAP). In order to be a recognized fellow, psychiatrists must pass a written and oral examination. However, the two countries differ in terms of training programs for child and adolescent psychiatry. In Japan, there is no national training program for CAPs, while there are three training programs for CAPs in the Philippines.

There is also a lack of psychologists in both countries. There are no child psychologists in Japan but there are many adult psychologists working in the field of child psychology. The Philippines also faces the challenge of having very few child psychologists in the country.

In addition to the lack of child psychiatrists and psychologists in both countries, Japan and the Philippines also lack teachers who can teach children with special needs (SPED teachers). Japan also faces increasing cases of *futoukou*, or children who refuse to go to school. School refusal is a complex problem and is possibly caused by several factors such as school bullying, trauma, and relationship issues. Meanwhile, in the Philippines, teachers who encounter children with behavioral problems conduct home visits to determine what kind of support, e.g. referral, the children and their family need. Some children drop out of school due to conduct problems.

Some differences in terms of the availability of mental health workers in the school setting were also noted. In Japan, all schools have a school nurse, majority have a school counselor, and some schools even have a social worker. The guidance counselors and teachers play a major role in detecting mental health problems among students and are trained to deal with mental health issues. In contrast, most of the public schools in the Philippines have nurses assigned at the division level (i.e. one nurse provides school health services for several schools). In addition, due to a lack of guidance counselors in public schools, some schools assign a school guidance teacher. However, private schools have their own school nurse and guidance counselor.

### Health financing

In Japan, the national health insurance provides 100% subsidy for inpatient and outpatient medical expenses of children below junior high school age (i.e. below 15 years old) care. After junior high school, medical expenses are partially subsidized by the government (70%) and the remaining costs will be out-of-pocket (30%). However, children sometimes need to wait for 3 months to a year to see a specialist due to the overcrowding of hospital CAP units. Financial resources from the welfare section of local governments are also available to provide support to families.

In the Philippines, majority of individuals with mental health disorders pay mostly or entirely out-of-pocket for services and medicines. However, inpatient care at government hospitals is free since the care and treatment of individuals with major mental disorders such as bipolar disorder, depression, and psychosis are covered by the national health insurance [7]. Nevertheless, the Philippine Health Insurance only reimburses the first week of confinement and it is selective about the diseases it covers. Moreover, it does not cover child mental health. Upon discharge from an inpatient facility, patients can avail of free medicines from the Department of Health’s Medicine Access Program. Patients can also apply to the Philippine Charity Sweepstakes Office (PCSO) which can cover at least 3 months’ worth of free medication provided that the medical doctor will give the medical abstract. Discounts can also be applied for persons with disability (PWDs) when they purchase medicines. Outpatient cases are not subsidized by the government and patients need to pay 100% of the cost from their own pocket.

### Pharmacotherapy

The same medications for ADHD, depression, and childhood depression are available in both Japan and the Philippines. Drugs for ADHD such as methylphenidate (MPH) and atomoxetine (ATX) and drugs for ASD such as risperidone (RSP) and aripiprazole (APZ) are being used in both countries. However, more medicines are available in Japan. For example, drugs such as amphetamine, guanfacine (GXR) and lisdexamfetamine (LDX) for the treatment of ADHD and pimozide for ASD are available in Japan but not in the Philippines. Unlike Japan, the Philippines does not use psychostimulants as first line drugs for ADHD treatment.

The Philippines follows the UK National Institute for Health and Care Guidelines (NICE) Clinical Guideline for Autism management for pharmacological treatment. It also emphasizes that treatment requires multidisciplinary action. The environment may play a role why children are exhibiting challenging behaviors hence it is recommended to address environmental factors prior to recommending medication.

In contrast with the Philippines, where off-label use of medicines is not commonly practiced, the off-label use of psychotropic drugs among children in Japan is common. Almost all Pharmaceuticals and Medical Devices Agency (PMDA)-approved drugs are authorized for use among children in Japan. The off-label use of antipsychotics is not associated with patient refusal of the prescription; rather, the most common factor for patient refusal of medications was the belief that antidepressant use causes more harm than good. Glucose and prolactin monitoring are infrequent in children initiated with antipsychotic therapy [21]. Concern in the use of antipsychotics in pediatric patients in Japan is also limited but there is a need for psychiatrists to routinely monitor the metabolic condition of patients. Additionally, standard educational programs and practice guidelines that provide evidence-based support to psychiatrists for prescription of psychotropic drugs are needed in Japan.

Both countries reported that a special license is needed by psychiatrists to prescribe certain stimulants such as methylphenidate. However, the prescription rate of MPH in Japan is lower than that in other countries, which may be associated with the restriction policy for prescribing stimulants in Japan.

### Psychosocial intervention

Both countries employed multidisciplinary teams to manage cases. The team is composed of child psychiatrists, social workers, nurses, and occupational therapists. For child abuse cases in both Japan and the Philippines, social workers serve as case managers.

Japanese CAPs are trained on different forms of psychotherapy during their training. Trauma-focused Cognitive Behavioral Therapy (TF-CBT), adapted from Cognitive Behavioral Therapy (CBT), is widely used for abused children in Japan. In the Philippines, some social workers are trained to conduct CBT-based therapy for child abuse cases.

Charging fees for psychotherapy are unclear for both countries. Psychotherapy provided by public Japanese facilities are free. In the Philippines, government hospitals with psychiatric facilities do not charge consultation fee. In some of these hospitals, there is an initial expense for the payment of a hospital ID. Expenses for laboratory examinations are paid for by the patient.

### Disaster child psychiatry

In times of disasters, children experience a wide range of mental and behavioral disturbances such as sleeplessness, fear, anxiety, depression, and post-traumatic stress disorder [22]. In Japan, children who were affected by the GEJE experienced long-term sleep disruption [23], with children from the affected Fukushima area exhibiting increasing numbers of suicide, child abuse, bullying, and absenteeism. Suicide risk and psychological symptoms were also observed among junior high school students 5 years after the GEJE. Children with evacuation experience and living in temporary housing had externalization symptoms. Economic disparities, the parents’ mental state and less social support may affect the children. In the Japanese experts’ experience, care for disabled children after disasters is also a challenge; children with ASD have difficulty adjusting to the crowded evacuation centers.

Evacuation centers in the Philippines are usually crowded after a disaster and this in turn, affect the mental health of the children and their family. Adding to this problem is the lack of mental health services for children in the Philippines after disasters. Due to the small number of practicing child psychiatrists in the country, adult psychiatrists have also been trained on how to deal with trauma of children after disasters and they examine child patients in some cases. Psychologists also help out during disasters. The Philippine Psychiatric Association also train people to process the trauma that children have experienced.

In the Philippines, where more than 90% of the total population identify as Christians, religion plays a major role in the social fabric and has become an important pathway for psychosocial support. Faith-based organizations have established mental health and psychosocial support services (MHPSS) especially during times of disasters, such as when Typhoon Haiyan struck in 2013 [24].

Mental health problems can impact children long after the disaster [25], hence providing mental health support is vital [26]. Following traumatic experiences such as disasters, schools, especially teachers, can play a key role in maintaining the well-being of children and adolescents [27, 28]. Psychological first aid is described as a “*humane, supportive response to a fellow human being who is suffering and who may need support*” [29]. In Japan and the Philippines, teachers undergo training on psychological first aid (PFA) and are being trained to identify children who are traumatized. Teachers are also trained on some play sessions and storytelling they can use with the children to help them deal with their trauma. In terms of psychological preparedness, Japan does not have psychological preparedness in schools. In the Philippines, while psychological preparedness is not integrated in the curriculum, some schools conduct trainings on psychological preparedness for teachers and students alike.

Differences were also observed in terms of government response to disasters. Concerted efforts by the Japanese government facilitated an efficient response to the needs of the population affected by the disaster. The Great Hanshin-Awaji Earthquake, which occurred in 1995, was the first disaster that focused on the need for mental health care for affected individuals. Interest in volunteer activities spiked in the aftermath of this disaster. In 2011, Kokoronokea (mental healthcare in Japanese) team provided medical support specializing psychiatry during the Great East Japan Earthquake (GEJE) and in 2013 the Disaster Psychiatric Assistance Team (DPAT) was established from this experience. The importance of providing support for carers or supporters and collaborating with educational institutions and school counselors were also vital lessons that Japan learned from the 2016 Kumamoto earthquake. In contrast, the Philippine experience during the aftermath of Typhoon Haiyan in 2013 highlighted the need for better coordination among non- government organizations as well as between these organizations and the government.

## Conclusion

The activities of the training program held in Japan and the Philippines successfully provided an opportunity to share the current situation on the care, diagnosis, and management of mental disorders in children and adolescents in the Philippines and Japan. In addition, the training program enabled Japanese and Philippine experts to identify similarities and differences and sharing of best practices between the two countries. The importance of creating partnerships with the religious sector was also highlighted. The training program is expected to create more opportunities for exchanging best practices on child and adolescent mental health promotion and care among countries in the future.

### Clinical implication and recommendation

Based on the outcome of the roundtable discussions, it is recommended to collaborate with the societies of other practitioners such as pediatricians, psychologists, teachers, and social workers to improve the identification and diagnosis of mental disorders. In addition, training other practitioners in identifying cases of mental disorders among children and adolescents can help ease the lack of child and adolescent psychiatrists in both countries.

Further studies on pharmacotherapy dosages specific to Asian setting needs to be done. In addition, developing clinical guidelines and protocols at the country or regional levels for treating children with mental disorders is also recommended. A standard system for availing of psychotherapy including its payment schemes will also be beneficial to children and families who avail of these services.

Cooperation between government efforts pre, during and post disasters is necessary to ensure that affected children and their families are provided with the needed and appropriate care and support. It is also important to provide long-term support to ensure the well-being of children and adolescents. Likewise, psychosocial preparedness needs to be integrated into school and community activities to equip the population with the knowledge and skills that are needed before, during, and after a disaster.

## Data Availability

Not applicable.

## Abbreviations

ADHD: Attention deficit hyperactivity disorder
APZ: Aripiprazole
ASD: Autism spectrum disorder
ASEAN: Association of South East Asian Nations
ATX: Atomoxetine
CAP: Child and adolescent psychiatry
CBT: Cognitive behavioral therapy
DALY: Disability-adjusted life years
DPAT: Disaster Psychiatric Assistance Team
FPS: First Person Shooting
GEJE: Great East Japan Earthquake
GXR: Guanfacine
JSCAP: Japanese Child and Adolescent Psychiatry Society
LDX: Lisdexamfetamine
LPC: Local Councils for the Protection of Children
MHGap: Mental Health Gap
MHPSS: Mental Health and Psychosocial Support Services
MPH: Methylphenidate
NCGM: National Center for Global Health and Medicine
PAP: Philippine Psychiatric Association
PCSO: Philippine Charity Sweepstakes Office
PFA: Psychological first aid
PPAEVAC: Philippine Plan of Action to End Violence Against Children
PSCAP: Philippine Society of Child and Adolescent Psychiatry
PTSD: Post-traumatic stress disorder
PWD: Persons with disability
RSP: Risperidone
TF-CBT: Trauma-focused cognitive behavioral therapy
WCPU: Women and Child Protection Unit
